# Urticarial Vasculitis in a Teenage Girl

**DOI:** 10.1177/2324709613484301

**Published:** 2013-04-12

**Authors:** Aaron McGuffin, Amy Vaughan, Juliet Wolford

**Affiliations:** 1 Marshall University Joan C. Edwards School of Medicine, Huntington, WV, USA; 2 UC Irvine Medical Center, Orange, CA, USA

**Keywords:** adolescent, urticarial vasculitis, urticaria, bias

## Abstract

This case involves a 13-year-old female who presented to the pediatrician for a routine check-up with complaints of a long history of intermittent diarrhea followed by a severe rash lasting for up to a week afterwards. The mother had described her daughter’s condition to multiple physicians, several whom had seen her during flare-ups. The nonmigratory lesions resembled “hives” with a single lesion lasting for 48 to 72 hours and resolving into what her parent described as a bruise. They often diagnosed her daughter with urticaria and prescribed steroids, which did resolve the acute flare-ups. None of the physicians, however, focused on the disease’s evolution and chronicity in an effort toward diagnosis and prevention. The patient was referred by her pediatrician to a dermatologist who diagnosed the patient with urticarial vasculitis. She was initially started on dapsone 25 mg and was increased over a period of months to a maintenance dose of 100 mg daily. She has had no recurrences in her cutaneous or systemic symptoms on this dose. She is closely monitored by her dermatologist on a regular basis with twice yearly complete blood counts. Several attempts have been made to discontinue the dapsone, resulting in a flare of her gastrointestinal symptoms. This patient suffered with this condition for almost 10 years. This is a reminder that spending extra time to think through a patient’s problem early on may prevent years of suffering for patients and their families.

## Introduction

Urticarial vasculitis (UV) is a multisystem disease characterized by cutaneous lesions resembling urticaria and biopsy findings of leukocytoclastic vasculitis. It can be accompanied by multiple symptoms, including varying degrees of arthritis, arthralgia, angioedema, uveitis, myositis, and abdominal or chest pain.^1^

Pediatric clinical practice involves a predominant mixture of common diagnoses with which pediatricians are intimately familiar: otitis media, asthma exacerbations, constipation, and so on. However, once every few years or perhaps once in a career, a disease we have never seen presents itself in the office. It is critical that the physician have the curiosity, drive, and lifelong learning skills to make the diagnosis.

Hives are common in pediatrics, but hives consistently preceded by diarrhea is clearly an atypical story. The temptation might be to treat the patient based on what we are familiar with, a pitfall known as “availability bias.” Availability is the tendency to reach for the plausible explanation nearest to hand and ignore competing theories. It is closely related to the error of “premature closure,” which is the tendency to stop considering other possible diagnoses after a diagnosis is reached. If the physician falls into either or both of these errors in thinking, the patient simply ends up with a prescription for steroids and a hope that the condition improves.

However, through a review of the patient’s paper and electronic medical health records, as well a review of the medical literature focusing on the unique diarrhea–hive connection, a diagnosis was made. The patient’s life was restored to normality, school absences were eliminated, and her mother was deeply grateful and relieved.

## Case Presentation

A 13-year-old female presented to the pediatric clinic with her mother who was a nurse at a local hospital. The patient complained of chronic intermittent diarrhea typically of 3 to 4 days duration, which was predictably followed by a painful, pruritic rash. The diarrhea, which always began at night, was profuse, watery, and nonbloody. It was associated with diffuse, crampy, abdominal pain and, occasionally, vomiting. The rash manifested as hives that covered her body completely. The episodes had occurred approximately 4 times a year since she was a young child. They had recently become more frequent and resulted in school absences. She denied fevers, cardiac, respiratory, or renal symptoms. There was no association with her menstrual cycle. She was otherwise healthy and took no chronic medications and had no recent antibiotics. She had no known allergies. There was no family history of dermatologic or gastrointestinal disease. She had no recent vaccinations. Loperamide had minimal effect on the diarrhea. The rash improved with diphenhydramine and steroids and usually resolved within a week.

Examination at the time of the visit revealed a well appearing female with blood pressure 112/52 mm Hg, pulse 68, and temperature 97.7°F. Head, eyes, ears, nose and throat (HEENT), cardiovascular, pulmonary, and abdominal exams were unremarkable. The patient has no evidence of rash or other abnormal skin conditions.

A dermatology referral was made and the patient was scheduled for lab work prior to the appointment. Before the visit to the dermatologist, the patient had a recurrent episode over a weekend. Her mother took her to the emergency room where she was evaluated and completed the recommended lab work. Results included normal electrolytes, liver function tests, blood urea nitorgen, creatinine, amylase, lipase, antinuclear antibody, tryptase, urinalysis, C3, and C4. Her white blood cell count was 18 900/mm^3^, hemoglobin 15.9 g/dL, and platelets 277 000/mm^3^. A CH50 was slightly elevated at 62 U/mL (normal = 22-60). A pregnancy test was negative. The patient received steroids and antihistamines in the emergency department with subsequent improvement.

**Figure 1.**
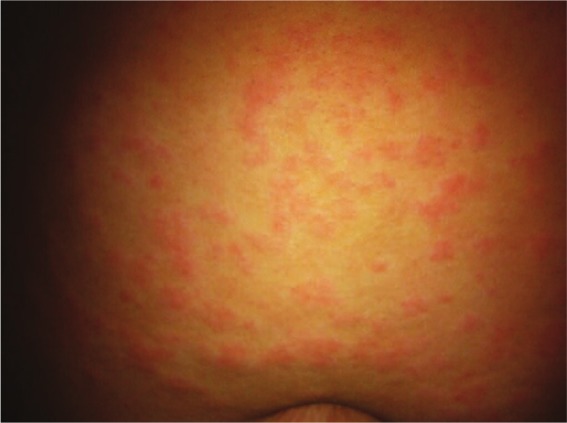
Diffuse wheals on the abdomen.

**Figure 2.**
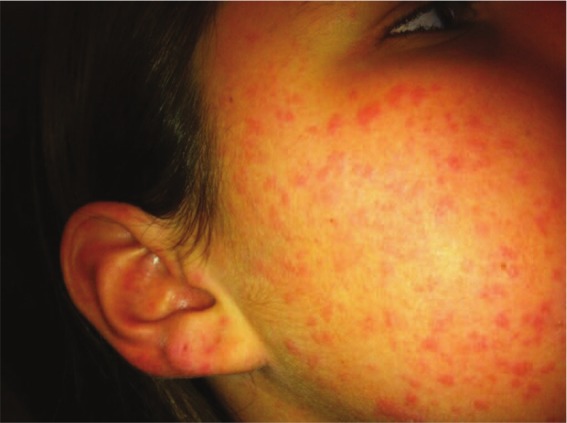
Right side of face with ear involvement.

**Figure 3.**
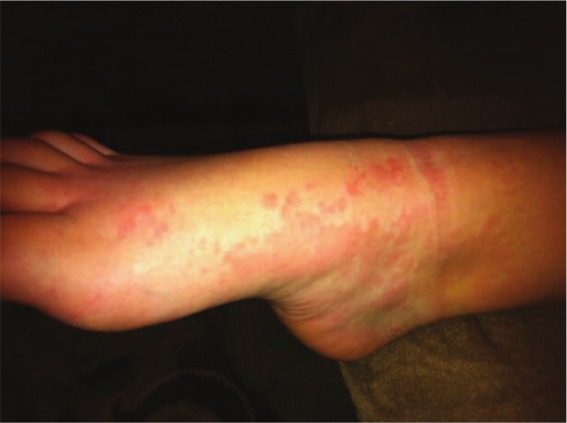
Right foot with coalescing urticaria.

Weeks later at the appointment with the dermatologist, the urticarial rash had faded. Based on a description of the symptoms, the pictures of the rash on the mother’s phone (see Figures 1-3), and the lab results, the patient was diagnosed with UV. A trial of dapsone 25 mg daily and loratadine 10 mg daily were initiated after discussing risks and benefits of pursuing therapy without a biopsy. A confirmatory biopsy was deferred due to the absence of lesions at the patient’s presentation to the dermatologist. Since the patient’s dose was increased to 100 mg daily, she has remained symptom free for more than 2 years.

## Discussion

Urticaria has numerous etiologies in the pediatric population. The most common causes include the following:

- Infections (viral, bacterial, parasitic)
- IgE-mediated allergies (medications, insects, foods, latex, blood products)
- Direct mast cell activation (radiocontrast agents, narcotics, vancomycin)
- Physical stimuli (cold, pressure, exertion, sun)

Pruritic wheals are typically the sole manifestation of the patient’s presentation and disappear within 24 hours, leaving no trace of their occurrence. The lesions of UV persist for at least 24 hours, thus differentiating them from typical urticaria. On resolution, there is usually purpura and hyperpigmentation on the involved skin. In UV, the patient’s urticaria involves painful, pruritic, erythematous wheals with occasional central clearing that can be found anywhere on the body.

UV is a rare condition that occurs most often in women in the fourth decade, although it can appear in children.^2^ Although the exact incidence and prevalence of UV are uncertain due to a lack of clarity in the medical literature resulting from the variety of cutaneous, systemic, and serologic features associated with the disease, the incidence of vasculitis in patients with apparent urticaria is between 5% and 20%.^3^

The disorder can be caused by viruses, rheumatic disease, or drugs but is most commonly idiopathic.^4^ Other systemic symptoms include angioedema, arthralgias, arthritis, photosensitivity, lymphadenopathy, cardiovascular disease, pulmonary disease, renal disease, episcleritis, uveitis, Raynaud phenomenon, diarrhea, abdominal pain, nausea, and vomiting.^4,5^

The presence of UV in the pediatric population is very rare, but it should be included in the differential in a patient who presents with symptoms that include an urticarial rash, kidney disease, pulmonary disease, and arthralgias or arthritis. It is important to distinguish urticarial vasculitis from acute urticaria, chronic urticaria, acquired angioedema, and erythema multiforme since cases of UV in this patient population are sometimes associated with more severe renal involvement.^6^

The etiology of UV is thought to be a type 3 hypersensitivity reaction in which immune complexes lodge in small blood vessel walls with activation of the complement pathway. This activation results in mast cell degranulation that causes urticarial eruption and neutrophil release of proteolytic enzymes that damage the vessel walls, leading to further tissue damage and edema.^5-7^

Diagnosis of UV is made in the setting of chronic urticaria with evidence of systemic symptoms. Histological demonstration via biopsy of small-vessel vasculitis, also known as leukocytoclastic vasculitis, is confirmatory.^2^ Patients with UV may have an elevated erythrocyte sedimentation rate, a positive antinuclear antibody, and hypocomplementemia.^5^

Treatment of UV is based on disease severity, extent of systemic involvement, and response to treatment. If the disease is mainly cutaneous, the treatment is symptomatic. Antihistamines can diminish itching, whereas glucocorticoids, dapsone, hydroxychloroquine, and colchicine can clear the rash. For systemic disease, indomethacin can be used to alleviate arthralgias and arthritis while glucocorticoids and dapsone can be used for the systemic symptoms. For severe refractory disease, immunosuppressive medications such as glucocorticoids in combination with azathioprine, cyclophosphamide, cyclosporine, or methotrexate can be used.^3-5,7^

Although UV is most often a benign self-limiting disease with an average disease interval lasting 3 to 4 years, it can, in some cases, last for decades.^2^ Because of the potential for lifelong debility, and treatability, it is essential to diagnosis this disorder as early as possible.

## Conclusion

In Dr Jerome Groopman’s book *How Doctors Think*, he points out that for emergency room physicians, anchoring on a diagnosis and availability are the 2 most frequent cognitive biases. And while “often they are all a doctor needs to hit the mark, to make a correct diagnosis and recommend an effective therapy . . . they can also veer wide of the mark.”^8^ For all physicians, our ultimate goal is to hit the diagnostic target in a timely and effective manner in order that we improve the lives of our patients. To accomplish this successfully, physicians must remain acutely self-aware of the quality of their history taking, the accuracy of their examination, and, most important, the critical thinking skills employed to make a final diagnosis, as ignoring vital pieces of the information may result in a decade of error.

## References

[1] Kane KSM Bissonette J Baden H Johnson R Stratigos A Color Atlas & Synopsis of Pediatric Dermatology. New York, NY: McGraw-Hill; 2002:361-362.

[2] Mehregan DR Hall MJ Gibson LE Urticarial vasculitis: a histopathologic and clinical review of 72 cases. J Am Acad Dermatol. 1992;26:441-448. 1564151 10.1016/0190-9622(92)70069-r

[3] Black AK Urticarial vasculitis. Clin Dermatol. 1999;17: 565-569. 10590850 10.1016/s0738-081x(99)00062-0

[4] Venzor J Lee W Hutson D Urticarial vasculitis. Clin Rev Allergy Immunol. 2002;23:201-216. 12221865 10.1385/CRIAI:23:2:201

[5] Davis MD Brewer JD Urticarial vasculitis and hypocomplementemic urticarial vasculitis syndrome. Immunol Allergy Clin North Am. 2004;24:183-213. 15120147 10.1016/j.iac.2004.01.007

[6] Hughes R Lacour JP Baldin B Reverte M Ortonneand JP Passeron T Urticarial vasculitis secondary to H1N1 vaccination. Acta Derm Venereol. 2010;90:651-652. 21057759 10.2340/00015555-0950

[7] Habif TP, ed. Clinical Dermatology. 5th ed. Philadelphia, PA: Mosby Elsevier; 2010:209-210.

[8] Groopman J How Doctor’s Think. New York, NY: Mariner Books; 2008:75.

